# Increased White Matter Inflammation in Aging- and Alzheimer’s Disease Brain

**DOI:** 10.3389/fnmol.2017.00206

**Published:** 2017-06-30

**Authors:** Divya Raj, Zhuoran Yin, Marjolein Breur, Janine Doorduin, Inge R. Holtman, Marta Olah, Ietje J. Mantingh-Otter, Debby Van Dam, Peter P. De Deyn, Wilfred den Dunnen, Bart J. L. Eggen, Sandra Amor, Erik Boddeke

**Affiliations:** ^1^Department of Neuroscience, Section Medical Physiology, University Medical Center Groningen, University of GroningenGroningen, Netherlands; ^2^Department of Neurology, Tongji Hospital, Tongji Medical College, Huazhong University of Science and TechnologyWuhan, China; ^3^Department of Pathology, VU University Medical CenterAmsterdam, Netherlands; ^4^Department of Nuclear Medicine and Molecular Imaging, University Medical Center Groningen, University of GroningenGroningen, Netherlands; ^5^Laboratory of Neurochemistry and Behavior, Institute Born-Bunge, University of AntwerpWilrijk, Belgium; ^6^Department of Neurology and Alzheimer Research Center, University Medical Center Groningen, University of GroningenGroningen, Netherlands; ^7^Biobank, Institute Born-BungeWilrijk, Belgium; ^8^Department of Pathology, University Medical Center Groningen, University of GroningenGroningen, Netherlands; ^9^Neuroimmunology Unit, Blizard Institute of Cell and Molecular Science, Barts and The London School of Medicine and DentistryLondon, United Kingdom

**Keywords:** white matter, microglia, neuroinflammation, aging, Alzheimer’s disease

## Abstract

Chronic neuroinflammation, which is primarily mediated by microglia, plays an essential role in aging and neurodegeneration. It is still unclear whether this microglia-induced neuroinflammation occurs globally or is confined to distinct brain regions. In this study, we investigated microglia activity in various brain regions upon healthy aging and Alzheimer’s disease (AD)-related pathology in both human and mouse samples. In purified microglia isolated from aging mouse brains, we found a profound gene expression pattern related to pro-inflammatory processes, phagocytosis, and lipid homeostasis. Particularly in white matter microglia of 24-month-old mice, abundant expression of phagocytic markers including Mac-2, Axl, CD16/32, Dectin1, CD11c, and CD36 was detected. Interestingly, in white matter of human brain tissue the first signs of inflammatory activity were already detected during middle age. Thus quantification of microglial proteins, such as CD68 (commonly associated with phagocytosis) and HLA-DR (associated with antigen presentation), in postmortem human white matter brain tissue showed an age-dependent increase in immunoreactivity already in middle-aged people (53.2 ± 2.0 years). This early inflammation was also detectable by non-invasive positron emission tomography imaging using [^11^C]-(R)-PK11195, a ligand that binds to activated microglia. Increased microglia activity was also prominently present in the white matter of human postmortem early-onset AD (EOAD) brain tissue. Interestingly, microglia activity in the white matter of late-onset AD (LOAD) CNS was similar to that of the aged clinically silent AD cases. These data indicate that microglia-induced neuroinflammation is predominant in the white matter of aging mice and humans as well as in EOAD brains. This white matter inflammation may contribute to the progression of neurodegeneration, and have prognostic value for detecting the onset and progression of aging and neurodegeneration.

## Introduction

Chronic neuroinflammation is a long-lasting inflammatory response that includes the persistent activation of local immune cells (microglia), the release of inflammatory molecules, and the enhancement of oxidative stress (Frank-Cannon et al., 2009). Gene expression studies in the aging brain have clearly outlined the importance of neuroinflammation and its role in neurodegenerative diseases (Lee et al., 2000; Lu et al., 2004; Glorioso and Sibille, 2011). Glia cells, particularly microglia, are the major source of expressed neuroinflammatory genes in the brain of aged mice. The expression of molecules involved in pattern recognition (Letiembre et al., 2007) and phagocytosis (Hickman et al., 2013) is increased in aged microglia. Aging microglia respond stronger to peripheral immune stimuli, alluded to as microglia priming (Perry et al., 2007; Sierra et al., 2007; Godbout et al., 2008; Raj et al., 2014).

Age is the primary risk factor for neurodegenerative diseases (e.g., AD and Parkinson’s disease) (Hung et al., 2010). Accumulating evidence suggests that microglia-induced neuroinflammation is a major contributor to the etiology of age-related neurodegeneration (Krstic and Knuesel, 2013; Heppner et al., 2015), rather than a passive response. The process of neurodegeneration is associated with chronic neuroinflammation in various neurodegenerative diseases (Frank-Cannon et al., 2009). However, our understanding of neuroinflammation is still limited with respect to its causes and progression. Detecting neuroinflammation at an early stage may become a key for finding the means of preclinical diagnosis and therapeutic interventions (Yankner, 2000).

Microglia display phenotypical diversity in different brain regions (Olah et al., 2011), and brain region-specific effects of aging on microglial gene expression have also been reported (Grabert et al., 2016). With increasing age, the number of HLA-DR/MHC II-positive microglia, particularly in the white matter, is increased compared to other brain regions (Ogura et al., 1994; Sheffield and Berman, 1998). This may influence the myelin loss, and lead to the eventual cognitive decline as observed in aged human and non-human primates (Ogura et al., 1994; Sheffield and Berman, 1998). It has been reported that iNOS immunoreactive microglia mediate increased protein nitration in the white matter of the aging non-human primate brain (Sloane et al., 1999). This might lead to a decrease in white matter integrity. In addition, upon aging, white matter was shown to contain complement-immunoreactive oligodendrocytes in association with activated microglia (Duce et al., 2006). Both CD11c-positive cells and CD3-positive T cells were particularly enriched in the white matter of the aged brain (Stichel and Luebbert, 2007), which indicates a crucial role of leukocytes in age-related response in the brain. These studies suggest that microglia-induced neuroinflammation might be more pronounced in white matter regions of the aging brain. Neuroinflammation has been directly implicated in cognitive decline (Ownby, 2010). White matter tracts in the brain are important for learning- (Hill, 2013; Fields, 2015) and information-processing (Fields, 2008). Neuroimaging studies revealed that age-related white matter alterations underlie cognitive decline (Bendlin et al., 2010). Considering the above-mentioned factors, a detailed regional characterization of microglia-induced neuroinflammation could provide insights into the initiation of neurodegeneration.

Here, we analyzed the nature of microglial activity through both the course of healthy aging and AD-related pathology to investigate whether microglial activity shows a regional difference between white matter and gray matter. Both human and mouse tissues were studied. Gene expression analysis revealed involvement of microglia in the pro-inflammatory response, phagocytosis, and lipid homeostasis related to brain aging. Morphological changes in microglia and phagocytosis marker expression were most prominent in white matter regions of the aging mouse brain. Using postmortem human brain samples as well as non-invasive PET analysis, we were able to demonstrate that the inflammatory activation of microglia starts in the white matter regions already in middle-aged subjects. Finally, the analysis of neuroinflammation in human EOAD and LOAD samples showed prominently increased microglial activity in white matter regions of EOAD brains, compared to young controls. These data provide evidence that increased microglia-induced neuroinflammation is predominant in the white matter of aging- and AD brains. It is tempting to speculate that neuroinflammation in the white matter may be used as an early marker for the prediction of cognitive decline during aging.

## Materials and Methods

### Animals

Young (2 and 4 months), middle age (13 months), and aged (24 and 27 months) male C57BL/6 and DBA/2J mice were purchased from Envigo. The animals were group-housed under standard conditions (a 12 h light-dark alternating cycle, constant temperature and humidity) and standard chow diet *ad libitum* (ab diets; Cat. No. 2103). All animal experiments were carried out in accordance with the European Directive (2010/63/EU) on the protection of animals used for experimental and other scientific purposes (Kilkenny et al., 2010). The protocols were approved by the Animal Experimentation Committee of the University Medical Center Groningen.

### Acute Isolation of Microglia from Adult Mouse Brain

Animals were sacrificed by means of saline perfusion under inhalation anesthesia with 4% isoflurane in oxygen. The brains were isolated and kept in ice-cold dissection solution (medium A: HBSS containing 0.6% glucose and 15 mM HEPES buffer, Gibco). For the isolation of microglia from white and gray matter regions, the forebrain and cerebellum were cut into approximately 1.5 mm thick coronal sections. Dissection was performed under magnifying glass with the tissue wet with medium A. Corpus callosum, cerebellar white matter were pooled from 2 to 3 animals of a particular age group and considered as one sample from that age group. From the collected tissue, microglia were isolated at high purity (>98%) using a discontinuous Percoll gradient (Vainchtein et al., 2014). All steps of the isolation and staining procedure were performed at 4°C. Briefly, the tissue was transferred to a tissue homogenizer (glass potter, Braun Melsungen, Germany), and mechanically dissociated. The brain homogenate was then filtered through a 70 μm cell strainer, washed with medium A, and pelleted by centrifugation (220 ×*g*, 10 min, arc 9, brake 9, 4°C). The density gradient separation was done using Percoll solutions with different densities (GE Healthcare, 17-0891). To obtain a stock isotonic Percoll solution (100%, density 1.123 g/ml), nine volume parts of Percoll (density 1.13 g/ml) were mixed with one volume part of 10x HBSS. Percoll solutions with the appropriate concentration were prepared via dilution of 100% Percoll with 1x PBS.

For the phagocytosis assay (*n* = 4) and FACS analysis (*n* = 5), the cell pellet was resuspended in 75% Percoll (10 ml), overlaid with 25% Percoll (10 ml), and PBS (6 ml) was added as the final layer. Density separation was achieved by centrifugating the discontinuous Percoll gradient in a swinging bucket centrifuge (800 ×*g* without brake for 25 min). After centrifugation, cells were collected from the 75–25% Percoll interface, washed with PBS, and pelleted by centrifugation (220 × *g*, 3 min, arc 9, brake 9, 4°C). The cell pellet was resuspended in culture medium containing DMEM without phenol red, and containing 5% FCS, 1% penicillin/streptomycin, and 1% sodium pyruvate for the phagocytosis assay. In case of flow cytometric analysis of surface expression markers, the pellet was resuspended in medium A which was prepared with HBSS devoid of phenol red. Subsequent sample preparation steps are described in the flow cytometry section.

For RNA used for microarray and quantitative real-time polymerase chain reaction (qPCR), the cell pellet was resuspended in 22% Percoll, and centrifuged for 20 min at 950 ×*g*. The pellet was resuspended in HBSS buffer, and incubated with CD11b-BV421 (Biolegend, 101236) and CD45-FITC (eBioscience, 11-0451-85). The CD11b^hi^/CD45^int^/DAPI^neg^ population was isolated using FACS (FACSAria III cell sorter, BD Biosciences). The collected populations were lysed in RLT lysis buffer in subsequent steps for preparing the microarray and qPCR.

### Flow Cytometry

Cells resuspended in medium A (without phenol red) were treated with 1% anti-CD16/32 (eBiosciences, 14-0161) for 15 min to block Fc receptors and subsequently stained for different surface markers (see below) for 20 min, washed with PBS, pelleted by centrifugation, and resuspended in 200 μl of medium A for subsequent flow cytometry analysis. During the staining procedure, cells were kept on ice. The surface expression of markers was measured with an FACSCalibur^TM^ flow cytometer (Becton Dickinson), and the flow cytometric measurements were analyzed using FlowJo software^®^. A small sample of the cell suspension was stained for CD11b and CD45, and the nuclear stain 4′,6-diamidino-2-phenylindole (DAPI, Biostatus), to demonstrate the vital microglia (CD11b^hi^/CD45^int^/DAPI^neg^), and to determine the purity of the preparation. The antibodies used for immunophenotyping of mouse microglia are listed in **Table 3**. For each staining, the appropriate isotype control was used in a concentration-matched manner.

### RNA Amplification, Microarray, and Analysis

Microglia (*n* = 8) were sorted as a CD11b^hi^/CD45^int^/DAPI^neg^ population in RNA lysis buffer, and RNA was extracted using the Qiagen RNeasy micro kit (Qiagen, 74004). RNA concentration and integrity were measured on the Experion RNA HighSens chip (Bio-Rad). Additional RNA amplification was performed with the Nugen Ovation RNA amplification kit. Subsequently, RNA was labeled and hybridized onto the Illumina mouseref-8 V2.0 expression beadchip containing 25,600 probes, coding for 19,100 genes. Genomestudio (version 1.9.0) was used to generate expression values. Raw data were preprocessed and analyzed using project R (version 2.13.1) and BioConductor package Limma (version 1.8.18). Background correction was done using infrared negative probes, and subsequently, quantile normalization and log_2_ transformation were applied. Probes were filtered out below a detection level of *p* < 0.05 in all samples as “no expression” cases. A linear model approach was used to perform differential gene expression analysis (with a FDR of *p* < 0.05 as the cut-off value for significance). For further GO and pathway analysis, DAVID (Database for Annotation, Visualization, and Integrated Discovery) were used. Heatmaps were generated using the heatmap.2 function of Bioconductor Package gplots.

### qPCR Experiments

For microarray validation, RNA from sorted microglia (*n* = 4) was extracted using the Qiagen RNeasy micro kit (Qiagen, 74004). Reverse transcription of the RNA was performed on a Mini^TM^ Thermal cycler (Bio-Rad) with a reaction mixture containing RevertAid^TM^ M-MuLV Reverse Transcriptase, Ribolock^TM^ RNase Inhibitor, and M-MLV buffer (all Fermentas). The RT-qPCR reaction, which contained iQ^TM^ SYBR^®^ Green Supermix (Bio-Rad, 170-8882), was performed in 384-well plates (Applied Biosystems) in an ABI7900HT Real-time PCR system (Applied Biosystems). All primers from Biolegio, were designed using NCBI Primer BLAST software. **Table 4** details the primers used. The other qPCR experiments were also performed in the same method using at least two housekeeping genes HMBS, HPRT in each experiment.

### Phagocytosis Assay

Isolated cell pellets were resuspended in culture medium, and seeded in an 8-well Lab-Tek^TM^ II Chambered Coverglass (Thermo Fisher Scientific, 155409) at the density of 5,000 cells per well. Cells were attached to the culture dish. Two hours after seeding, the medium was replaced with culture medium containing 25 μg/ml of pHrodo^TM^ E. coli BioParticles^®^ conjugate (Thermo Fisher Scientific, P35366). The cells were subsequently imaged for 18 h with a *Solamere Nipkow* S*pinning* Disc Confocal laser scanning microscope. The microscope was mounted on a Leica DM IRE2 inverted microscope which was equipped with a Stanford Photonics XR/Mega-10I (intensified) CCD camera and an ASI MS2000 Piezo motorized stage (37°C, 5% CO2). The pHrodo dye was excited with the 568 nm laser line of a dynamic Krypton laser. For image acquisition, a 10× dry objective was used. The fluorescence emission maximum of pHrodo is 585 nm. A bright field and a red channel image (pHrodo emission) were acquired every 5 min at each condition. Multiple cells were selected as ROIs based on the bright field images. The intensity of the ROIs in the red channel images was measured in each frame of the entire image stack using an ImageJ plugin (written by K. Sjollema; Microscopy Centre, University Medical Center Groningen, University of Groningen). The data were plotted as a time versus intensity curve, on which the time to reach half maximum response for each cell was determined.

### Immunohistochemistry for Animal Brain Tissues

Animals (*n* = 3) were anesthetized and were perfused transcardially for 20 min with 0.1M phosphate buffer (40–60 ml; pH 7.4). Brains were removed and cut into two sagittal parts. The right hemisphere was snap-frozen in liquid nitrogen. The left hemisphere was immersed in 4% PFA overnight at 4°C, then transferred to 25% sucrose in PBS for 1 day, and finally frozen at -50°C.

Brain sections (60 μm free-floating cryostat sections) from PFA-fixed samples were used for detecting IBA1 or Mac-2. Sections were incubated for 10 min in 2% H_2_O_2_ in 70% methanol, followed by blocking solution containing 5% normal goat serum or fetal calf serum in phosphate buffered saline (PBS) containing 0.1% Triton-X (Sigma, X-100) (denoted as PBS+) for 30 min at room temperature. Subsequently, sections were incubated overnight at 4°C with rabbit anti-IBA1 (1:1000; WAKO, 019-19741) or rat anti-Mac-2 (1:1000, Cedarlane, CL8942AP) (detailed information in **Table 5**). As negative controls, sections were incubated in buffer lacking the primary antibody. After washed with PBS+, sections were incubated at room temperature for 1.5 h with biotinylated goat anti-rabbit (1:200; Vector Laboratories, BA-1000) or biotinylated rabbit anti-rat (1:200; Vector Laboratories, BA-4000). Sections were then rinsed in PBS+, and incubated with streptavidin-horseradish peroxidase in accordance to the manufacturer’s instructions (PK-6100; Vector Laboratories). The peroxidase reaction was visualized by incubating the sections in PBS containing 0.5 mg/ml 3,3’-diaminobenzidine (DAB, Sigma, D-5637), and 0.33 μl/ml H_2_O_2_. Then free-floating sections were subsequently mounted on glass slides, dehydrated, and mounted with DePeX (Merck, 130-12-2). Alternatively, to differentiate between white and gray matter tissues, some sections were stained with Luxol Fast Blue (LFB) after the DAB reaction. Sections were subsequently mounted on glass slides and dehydrated in an ascending ethanol series (up to 96% ethanol), incubated in LFB working solution [prepared by dissolving 0.5 g of Solvent Blue 38 (Sigma, 229342) in 500 ml 96% ethanol including 10% acetic acid (Merck, 10063)] at 60°C overnight, and washed with 96% ethanol and distilled water. Differentiation was achieved in 0.125% lithium carbonate solution. The sections were rinsed in 70% ethanol followed by distilled water. After dehydration, the sections were mounted in DePeX. Slides were scanned using a ScanScope XT Digital Slide Scanner (Aperio) at a resolution of 0.25 mm/pixel (100,000 pix/in) and data was analyzed with image processing in Fiji. First we employed a color deconvolution method to separate stains of cresyl violet and LFB from DAB immunostaining, so that quantification of individual stains avoided cross contamination. The deconvoluted images were color-thresholded to mark the immunostained region within the image and positive pixels quantified on a particle analysis plugin. For cerebellum sample, white/gray matter could be hand drawn using free form regions and pixels quantified within these regions and normalized for area covered. A minimum of four brains were analyzed for each age.

Brain sections (10 μm) from snap-frozen samples were used for immunohistochemical analysis using the following primary antibodies: Axl, Dectin1, Trem2, CD36, CD16/32, and CD11c (detailed information in **Table 5**). Sections were fixed with acetone for 10 min and then air-dried. Endogenous peroxidase was inactivated by a 30-min incubation with peroxidase blocking reagent from the DAKO envision kit (DAKO, K4009). Sections were incubated for 30 min in 5% serum blocking, and then for 2 h in the primary antibody at room temperature, followed by rinsing with PBS. For those primary antibodies which were not raised in rabbit, sections were incubated with the secondary antibody which was raised in rabbit for another hour. Afterward sections were incubated with labeled polymer-HRP anti-rabbit (DAKO, K4009) at room temperature for 30 min. The complex was visualized after 10 min incubation with 3-amino-9-ethylcarbazole (AEC) substrate-chromogen solution (DAKO, K4009), and counterstained with Mayer’s hematoxylin (Merck, 104302). Sections were finally covered with glycerol jelly.

### Postmortem Human Brain Tissues

Postmortem human brain samples used for studying the aging effect were obtained from the Department of Pathology, VU University (Amsterdam). The rapid autopsy regimen of the Netherlands Brain Bank in Amsterdam (coordinator Dr. I Huitinga) was used to acquire subcortical white matter samples from 15 control donors without any known neurodegenerative condition (details in **Table 1**), with the approval of the medical ethical committee of the VU University Medical Center, and specific approval for the present study. All patients and control donors had given informed consent for autopsy and the use of brain tissue for research purposes.

**Table 1:** Patient data.

Case	Age (years)	Category in experiment	Disease/cause of death
1	30–35	Young	Subarachnoid bleeding
2	30–35	Young	Familial cardiac problems (No pathology in the brain)
3	25–30	Young	Arrhythmia (No pathology in the brain)
4	50–55	Middle-aged	Cardiac arrest (No pathology in the brain)
5	50–55	Middle-aged	Myocardial infarction, in brain minor hypertension related pathology
6	50–55	Middle-aged	Myocardial infarction
7	50–55	Middle-aged	Suicide
8	50–55	Middle-aged	Esophageal cancer/euthanasia
9	56–60	Middle-aged	Unknown
10	70–75	Aged	Gastrointestinal bleeding, no pathology in brain
11	70–75	Aged	Respiratory insufficiency due to bronchitis, in brain minor hypertension related pathology
12	70–75	Aged	CREST syndrome, in brain small lacunar infarcts in thalamus
13	85–90	Aged	Rectum and prostate cancer, cachexia and dehydration
14	80–85	Aged	Pleuritis carcinomatosis
15	85–90	Aged	Pneumonia and heart failure

Postmortem human brain samples used for investigating the effect of AD pathology were obtained from Pathology division of the Department of Pathology and Medical Biology, University Medical Center Groningen (Groningen) and the Biobank of the Institute Born-Bunge, University of Antwerp (Antwerp). Paraffin-embedded samples were classified according to Braak staging and age: (1) LOAD group: NFT stage = V or VI, age > 60 years, *n* = 4; (2) Old control group: old clinically silent cases, NFT stage = I, age > 60 years, *n* = 3; (3) EOAD group: NFT stage = V or VI, age ≤ 60 years, *n* = 5; (4) Young control group: young normal and clinically silent AD cases, NFT stage = 0 or I, age ≤ 60 years, *n* = 13 (details in **Table 2**). All donors have given written informed consent for autopsy and use of their brain tissue for research purposes. The use of samples from young and old controls and LOAD donors (details in **Table 2**) were approved by the medical committee of University Medical Center Groningen. The use of samples from 5 EOAD donors (details in **Table 2**) were approved by ziekenhuis network Antwerpen (ZNA) ethics committee.

**Table 2:** Patient data.

Sample ID	Age (years)	Braak stage	Category in experiment	Disease/cause of death
16	40–45	I	Young control	Arrhythmia
17	46–50	I	Young control	Cardiac death
18	46–50	I	Young control	Lymphoma
19	20–25	0	Young control	Cardiac death
20	30–35	I	Young control	Myocardial infarction
21	20–25	I	Young control	Pulmonary embolism
22	40–45	I	Young control	Septicemia (acute inflammation)
23	36–40	I	Young control	Cardiac death
24	36–40	I	Young control	Cardiac death
25	40–45	I	Young control	Drug poisoning
26	4–45	0	Young control	Cardiac death
27	20–25	0	Young control	Ulcerative colitis
28	40–45	I	Young control	Pancreatitis (acute inflammation)
29	36–40	I	Young control	Pneumothorax
30	30–35	I	Young control	Drug overdose
31	30–35	VI	EOAD	Familial AD (neuropathologically confirmed)
32	40–45	VI	EOAD	Familial AD (neuropathologically confirmed)
33	56–60	V–VI	EOAD	AD, mutation PSEN 1 gene
34	56–60	V–VI	EOAD	AD
35	56–60	V–VI	EOAD	AD
36	66–70	I	Old control	Sepsis, pericarditis (acute inflammation)
37	60–65	I	Old control	Pneumonia (acute inflammation)
38	80–85	I	Old control	Myocardial infarction
39	76–80	I	Old control	Pancreas carcinoma
40	76–80	I	Old control	Respiratory insufficiency
41	80–85	V	LOAD	Cardiac death, leg infection (acute inflammation)
42	60–65	VI	LOAD	Bronchopneumonia (acute inflammation)
43	76–80	V	LOAD	Cachexia
44	70–75	V	LOAD	Aortic rupture
45	70–75	VI	LOAD	Pancreas carcinoma
46	76–80	V	LOAD	Pneumonia (acute inflammation)
47	70–75	V	LOAD	n.k

**Table 3:** Antibodies for flow cytometry.

Antigen	Species reactivity	Host species	Vendor	Catalog number	Isotype	Conjugate
CD11b	Mouse	Rat	eBioscience	12-0112	Rt IgG2b	PE
CD45	Mouse	Rat	eBioscience	11-0451	Rt IgG2b	FITC
F4/80	Mouse	Rat	Biolegend	123107	Rt IgG2a	FITC
CD14	Mouse	Rat	eBioscience	11-0141	Rt IgG2a	FITC
Tlr1	Mouse	Rat	eBioscience	12-9011	Rt IgG2a	PE
Tlr4	Mouse	Rat	eBioscience	12-9041	Ms IgG1	PE
CD80	Mouse	Hamster	eBioscience	12-0801	Hm IgG	PE
CD83	Mouse	Rat	eBioscience	11-0831	Rt IgG1	FITC
MHC II	Mouse	Rat	eBioscience	11-5321	Rt IgG2b	FITC
CD36	Mouse	Rat	eBioscience	12-0361	Rt IgG2a	PE
CD88	Mouse	Rat	Biolegend	135805	Rt IgG2b	PE
Isotype	Mouse	Rat	Biolegend	407105	Rt IgG2a	FITC
Isotype	Mouse	Rat	Biolegend	400607	Rt IgG2b	PE
Isotype	Mouse	Rat	eBioscience	11-4210	Rt IgG2a	FITC
Isotype	Mouse	Rat	eBioscience	11-4220	Rt IgG2b	FITC
Isotype	Mouse	Rat	eBioscience	12-4015	Ms IgG1	PE
Isotype	Mouse	Hamster	eBioscience	11-4888	Hm IgG	PE

**Table 4:** Primer information.

Gene	Accession number	Forward sequence	Reverse sequence	Amplicon
*Itgax*	NM-020008	CCCAACTCGTTTCAAGTCAG	AGACCTCTGATCCATGAATCC	81 bp
*LgalS3*	NM-010705	CAGGATTGTTCTAGATTTCAGGAG	TGTTGTTCTCATTGAAGCGG	73 bp
*Axl*	NM-001190974	TGAAGCCACCTTGAACAGTC	GCCAAATTCTCCTTCTCCCA	117 bp
*CD36*	NM-001159558	GATGTGGAACCCATAACTGGA	AGGTACAATGTAAGGTCTCTTCAG	122 bp
*Apoe*	NM-009696	TGTGGGCCGTGCTGTTGGTC	GCCTGCTCCCAGGGTTGGTTG	106 bp

**Table 5:** Antibodies for Immunohistochemistry.

Antigen	Species reactivity	Host species	Vendor	Catalog number	Concentration
IBA1	Mouse/Human	Rabbit	Wako	019-19741	1:1000
Mac-2	Mouse	Rat	Cedarlane	CL8942AP	1:1000
Axl	Mouse	Goat	Santa Cruz	SC-1096	1:100
Dectin1	Mouse	Rat	AbD Serotec	MCA2289	1:100
CD36	Mouse	Rat	eBioscience	14-0361	1:100
CD16/CD32	Mouse	Rat	eBioscience	14-0161	1:100
CD11c	Mouse	Hamster	eBioscience	14-0114	1:100
Trem2	Mouse/Human	Rat	R&D systems	MAB17291	1:100
CD68	Human	Mouse	Dako	IR613	1:50
HLA-DR	Human	Mouse	eBioscience	14-9956-80	1:250

Brain samples from patients who died of acute inflammatory diseases (e.g., sepsis, pancreatitis) were used as positive controls for detecting neuroinflammation. Seven brain samples were obtained from the Pathology division of the Department of Pathology and Medical Biology, University Medical Center Groningen (details in **Table 2**). All donors had given written informed consent for autopsy and use of their brain tissue for research purposes. The use of samples were approved by the medical committee of University Medical Center Groningen.

### Immunohistochemistry for Human Brain Tissues

Paraffin-embedded tissue (5 μm) of human brains from AD and control groups were immunostained with IBA1 (1:1000, WAKO, 019-19741), CD68 (1:50, DAKO, M0876) and HLA-DR (1:100, eBioscience, 14-9956-82). Paraffin-embedded tissues from different age groups were immunostained with CD68 (1:100, DAKO, M0814) and HLA-DR (1:100, eBioscience, 14-9956-82). Sections were deparaffinized with xylene and rehydrated gradually from 100% ethanol to demi water. For antigen retrieval, the sections were placed in 10 mM sodium citrate buffer (pH 6.0) in a microwave for 12 min.

After rinsing in PBS, sections were pre-incubated in 3% H_2_O_2_ for 30 min and then blocked with 10% normal horse serum in PBS with 0.3% Triton-X100 for 30 min. Sections were incubated overnight at 4°C with abovementioned primary antibodies in PBS with 0.3% Triton-X100 and 1% normal horse serum. Sections were incubated with horse anti-mouse biotinylated antibody (1:400, Vector Laboratories, BA2001) for 1 h at room temperature, incubated in avidin-biotin-peroxidase complex for 30 min, and then visualized with DAB. Sections were counterstained with Cresyl Violet and mounted with DePeX.

Slides were scanned using a digital slide scanner (Hamamatsu), and data were analyzed with the positive pixel count algorithm (Imagescope). For each human sample, 5–10 pictures per brain region (20× magnification) were quantified. Transentorhinal region is the first region where the AD pathology evolves (Braak stage I), whereas frontal cortex (FC) is involved in the late stage of AD (Braak stage V) (Braak et al., 2006). To investigate the regional differences in AD and control groups, the positive pixels in the white matter below the transentorhinal cortex (EC) and in the FC were quantified. For samples from different age groups, the cortex and cerebellum were quantified. The pictures of the stained sections were separated by using color deconvolution method (Image J), and the DAB-positive pixels were quantified.

### Human Non-invasive PET Study for [^11^C]-(R)-PK11195 Binding

Seven young (25.1 ± 2.9 years) and seven middle-aged (55.7 ± 11.2 years) healthy subjects were included in the study. Exclusion criteria were the presence of inflammation as measured by CRP (i.e., CRP < 0.5 mg/L), concomitant or past severe medical conditions, substance abuse, use of non-steroidal anti-inflammatory drugs or paracetamol, and pregnancy. The study was approved by the Medical Ethical Committee of the University Medical Center Groningen. All subjects provided written informed consent after receiving a complete description of the study. All subjects had a structural T1-weighted MRI scan of the brain (1.5 or 3 T) within 2 weeks of the PET procedure. The MRI was used for anatomical reference and the delineation of the ROIs.

[^11^C]-PK11195 PET images were acquired using an ECAT EXACT HR+ camera (Siemens/CTI, Knoxville, TN, United States). A 60-min dynamic scan in 3D-mode was performed, which consists of 21 successive frames of increasing duration (6x 10, 2x 30, 3x 60, 2x 120, 2x 180, 3x 300, and 3x 600 s). During the scan, arterial blood radioactivity was continuously monitored with an automated sampling system, and additional manual arterial samples were taken for radio-metabolite analysis. Detailed scanning and sampling procedures were published previously (Doorduin et al., 2009). Head movement was minimized with a head-restraining adhesive band, and a neuroshield was used to minimize the interference of radiation from the subject’s body. The images were reconstructed by filtered back projection, and attenuation correction was performed with the separate ellipse algorithm. The PET and MRI images were coregistered using Statistical Parametric Mapping (SPM2) software, and the aligned MRI image was normalized to the SPM2 MRI template. The normalization was then applied to the PET images. ROIs were created using the automated anatomical labeling template (Tzourio-Mazoyer et al., 2002) or were manually drawn onto the MRI. The ROIs include the FC, occipital cortex, parietal cortex, temporal cortex, cerebellum, hippocampus, thalamus, basal ganglia, mesencephalon, pons, and corpus callosum. The time-activity curves of all ROIs were used for kinetic modeling using software developed in Matlab 7.1 (Mathworks, Natick, MA, United States). Two-tissue compartment modeling was used to calculate the K_1_–K_4_ according to the curve of whole blood and metabolite-corrected plasma. The primary outcome measure was the BP_P_, defined as k_3_/k_4_. Statistical analysis was performed in PASW Statistics 18. One-way ANOVA was used to determine differences in BP_P_ between young and middle-aged subjects. The correlation between age and the BP_P_ were assessed with Pearson’s product moment correlation coefficient (r). Differences were considered statistically significant when *p* < 0.05.

## Results

### Microglia in the Aged Mouse Brain Are Characterized by Increased Phagocytosis and Altered Lipid Homeostasis

To examine age-associated changes in gene expression, microglia were isolated from aged mouse brain, and RNA expression was determined. Microarray analysis of pure microglia showed > 1.5-fold increased expression of 54 transcripts in aged mouse microglia compared to young microglia. To relate the changes in gene expression to a biological function, we applied DAVID software and identified that differentially expressed genes were involved in biological categories such as antigen processing and -presentation, interferon signaling, regulation of macrophage cytokine production, chemotaxis, cell adhesion, phagocytosis of apoptotic cells, and lipid homeostasis.

**FIGURE 1:**
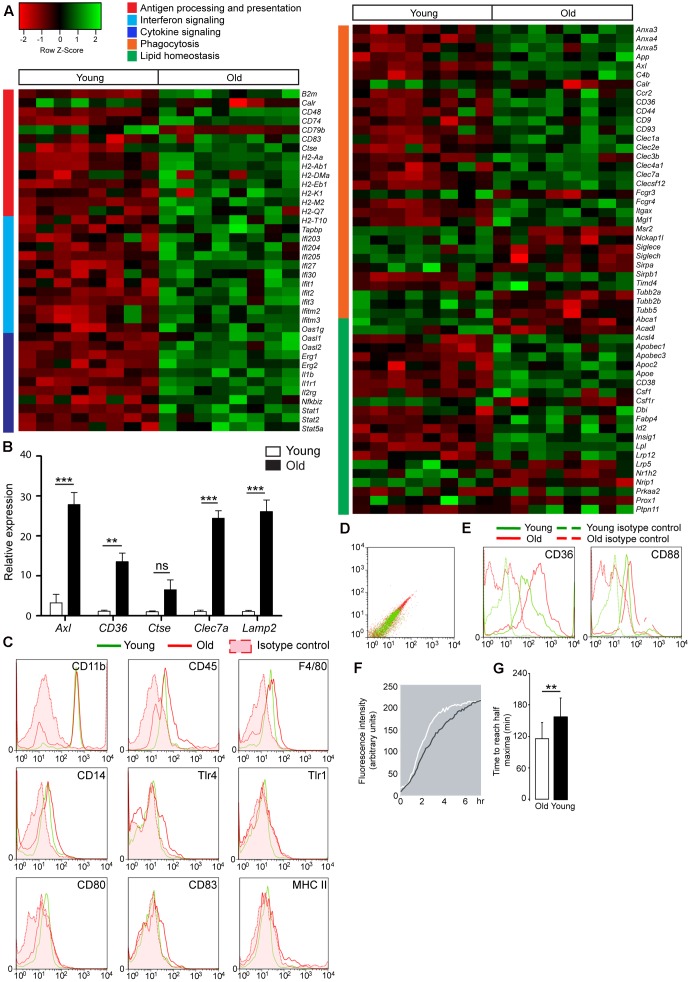
Gene expression change and increased phagocytic capacity in aged microglia, compared to young microglia. **(A)** Heatmap of pro-inflammatory genes in aged microglia vs. young mouse microglia. Functional annotation reveals genes upregulated in aged microglia to be involved in antigen processing and presentation (red cluster), interferon signaling (light blue cluster) and cytokine signaling (dark blue cluster) (*n* = 8); Heatmap of genes involved in phagocytosis (orange cluster) and lipid homeostasis (green cluster) in aged microglia vs. young microglia (*n* = 8); **(B)** Quantitative PCR validation of phagocytic genes in sorted microglia from young (white bars) and aged (black bars) mouse brain (*n* = 4 young; *n* = 6 old); HMBS was used as the housekeeping gene. Asterisks ^∗^ indicate comparisons, for which *P-*value was values indicated according to Student’s *t*-test, ^∗∗^*P* < 0.005, ^∗∗∗^*P* < 0.0005, ns, not significant. Error bars indicate standard deviation (SD). **(C)** After purification, gated cells are CD11b high, CD45 intermediate, and F4/80 positive microglia. Antigen presenting molecules, CD14, Tlr4, Tlr1, CD80, CD83, and MHC II, are not higher expressed at the protein level in aged microglia (*n* = 5); **(D)** Increased autofluorescence of aged microglia; **(E)** Lipid-related scavenger receptor CD36 and complement receptor CD88 are found to be upregulated in aged microglia (red) compared to young microglia (green) and higher than the corresponding isotype controls and autofluorescence values (derived from unstained cells) (*n* = 5); **(F)** The phagocytic capacity of acutely isolated microglia was investigated by means of live cell imaging using pHrodo coupled to bacterial particles (*n* = 4); **(G)** Quantification of the time to reach half maxima during phagocytic response of acutely isolated microglia from young and aged mouse brains. The aged microglia need less time to reach the half maxima. Each depicted experiment is representative of four independent experiments that yielded similar results. Student’s *t*-test ^∗∗^*P* < 0.005, Error bars indicate standard deviation.

Increased expression of genes in aged microglia, indicative of a pro-inflammatory status, belonged to categories of antigen presentation, interferon signaling, and cytokine signaling. These groups were represented as a heatmap (**Figure 1A**), and included several histocompatibility two genes (e.g., *Egr1*, *Egr2*, *Stat1*, and *Stat2*) and phagocytic receptor genes (e.g., *Axl*, *Clec7a*, *CD36*, *Clec1a*, *Fcgr4*, *Clec3b*, *Clec4a1*, *Anxa3*, *Anxa4*, and *Anxa5*). Phagocytosis-associated genes were strongly upregulated in aged microglia as validated by quantitative PCR of *Axl*, *CD3*6, *Ctse*, *Clec7a*, and *Lamp2* (**Figure 1B**). In addition, aged microglia showed changes in genes involved in cellular lipid homeostasis (e.g., *Apo*e, *Csf1*, and *Lpl*). The altered genes are depicted in a heat map (**Figure 1A**).

Despite their increased mRNA levels, the protein expression levels of pattern recognition receptors (e.g., CD14, Tlr1, Tlr4) and proteins required for antigen presentation (e.g., MHC II, CD80, and CD83) in aged microglia were not upregulated compared to young microglia as shown in **Figure 1C**. Acutely isolated microglia from aged mice display high autofluorescence levels compared to young microglia as shown by flow cytometry (**Figure 1D**). This could be due to the presence of lipofuscin. Aged microglia also upregulate the expression of lipid-related phagocytic receptor CD36 and complement receptor CD88 at the protein level (**Figure 1E**).

To study their phagocytic activity, acutely isolated microglia from young and aged mouse brains were incubated with pHrodo-coupled *Escherichia coli* (*E. coli)* bacterial particles. pHrodo is a pH sensitive dye which fluoresces only at acidic pH, as found in late endocytic vesicles. This allows live cell imaging of phagocytic uptake. Aged microglia phagocytose *E. coli* bioparticles faster than young microglia. This is likely mediated by increased expression of phagocytic receptors (**Figure 1F**). Quantification shows that aged microglia reach half-maximal levels of phagocytosis several minutes earlier than young microglia (**Figure 1G**).

### Increased Microglial Activity in the White Matter Tracts of the Aged Mouse Brain

**FIGURE 2:**
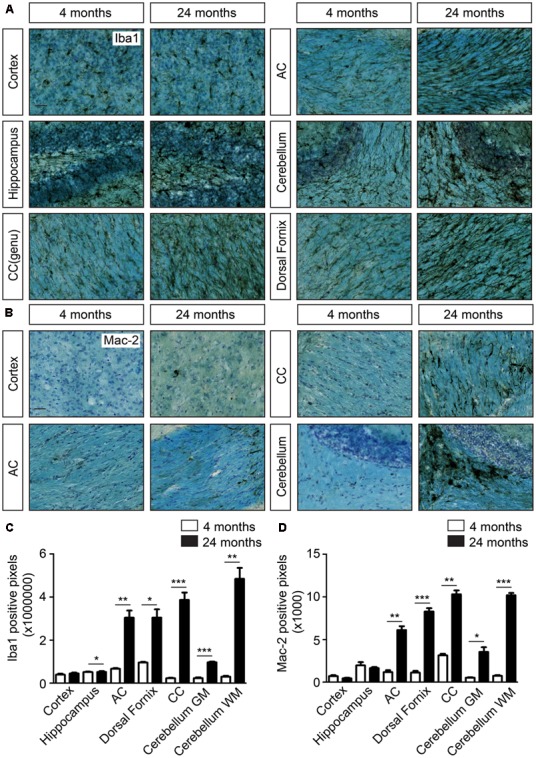
Increased cellular clustering and IBA1 immunoreactivity in the white matter of the aging brain. **(A)** IBA1 staining coupled to Luxol fast blue staining in young and aged brains. Comparison of the density of microglia in the cortex, hippocampus and white matter regions, such as corpus callosum (CC), anterior commissure (AC), cerebellar white matter, and dorsal fornix; **(B)** Mac-2 staining together with luxol fast blue staining show increased Mac-2 positivity in the white matter of aged compared to young brain. Increased Mac-2 positivity is observed in white matter tracts including corpus callosum, anterior commissure, and cerebellar white matter; **(C**) Quantification of IBA1 positive pixels in different regions of young (*n* = 4) and old brains (*n* = 4). **(D)** Quantification of IBA1 positive pixels in different regions of young (*n* = 4) and old brains (*n* = 4). Scale bar: **(A,B)** = 25 μm. For, **(C,D)** Student’s *t*-test ^∗^*P* < 0.05, ^∗∗^*P* < 0.005, ^∗∗∗^*P* < 0.0005, ns, not significant. Error bars indicate standard deviation (SD).

To investigate the effect of aging on the microglia phenotype, age-related changes in microglia morphology were visualized by staining with IBA1. During aging, IBA1 immunoreactivity particularly increased particularly in white matter regions, such as corpus callosum (2, 13, 24, and 27 months; Supplementary Figure 1A). Morphological changes were also visible in gray matter regions of the brain at 27 months (Supplementary Figure 1A). Microglia cell clusters and changes in microglia morphology were clearly observed at 24 months, particularly in white matter areas like corpus callosum, anterior commissure, dorsal fornix, and cerebellar white matter, compared to 4-month-old mice (**Figure 2A**). In addition to white matter-enriched regions, the cortex, hippocampus (**Figure 2A**), hypothalamus, and spinal cord gray matter regions (data not shown) also showed changes in microglia morphology in aged (24 months) mouse brain. Microglia cell clusters (Supplementary Figure 1B) and the presence of beaded structures in processes of IBA1-positive microglia (Supplementary Figure 1B) were exclusively found in the aged (24 months) mouse brain.

**FIGURE 3:**
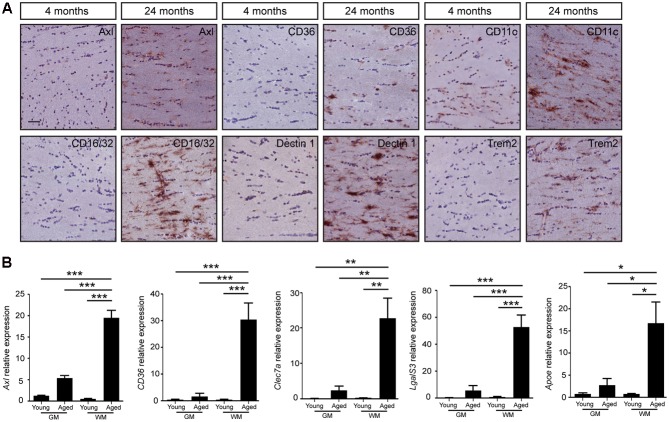
The change of microglial gene expression during aging mainly occurs in the white matter of mouse brain. **(A)** The increased expression of genes involved in microglia phagocytosis and activation (Axl, CD36, CD16/32, CD11c, Trem2, and Dectin1) is found specifically in the white matter of aged brain (24 months, *n* = 3), compared to young brain (4 months, *n* = 3); **(B)** Quantitative PCR evaluation of microglia phagocytic genes associated with aging (4 months, *n* = 4; 24 months, *n* = 4). Increased expression of phagocytic markers (*Axl*, *CD36*, *Clec7a*, *LgalS3*, and *Apoe*) in microglia isolated from white matter enriched brain regions. GM, gray matter; WM, white matter. HMBS was used as the housekeeping gene. ANOVA ^∗^*P* < 0.05, ^∗∗^*P* < 0.005, ^∗∗∗^*P* < 0.0005. Error bars indicate standard deviation (SD).

Mac-2, also known as *LgalS3*, expressed by phagocytic subpopulations of microglia (Rotshenker, 2009), was selectively detected in the white matter tracts of the aged brain, particularly in the corpus callosum, anterior commissure, and the cerebellar white matter (**Figure 2B**). Gray matter regions, such as cerebral cortex did not express Mac-2 (*LgalS3*, **Figure 2B**). Quantification of IBA1 (**Figure 2C**), Mac-2 (**Figure 2D**) immunoreactive pixels showed significant differences in number of positive pixels, particularly in white matter regions with aging. Similar patterns of expression were also observed for Axl, CD36, CD16/32, CD11c, Trem2, and Dectin1 (*Clec7a*) in the genu of corpus callosum (**Figure 3A**). All these proteins were highly expressed in the white matter of the aged brain. Microglia were isolated from white or gray matter-enriched brain regions, and checked for RNA expression of phagocytosis- and activation markers found in weighted correlation network analysis (WGCNA) (Holtman et al., 2015). Expression of *Axl*, *CD36*, *Clec7a*, *LgalS3*, and *Apoe* were found to be significantly higher in the white matter of the aged mice (**Figure 3B**).

### Neuroinflammation in the Human Brain Starts during Middle Age in White Matter Regions

**FIGURE 4:**
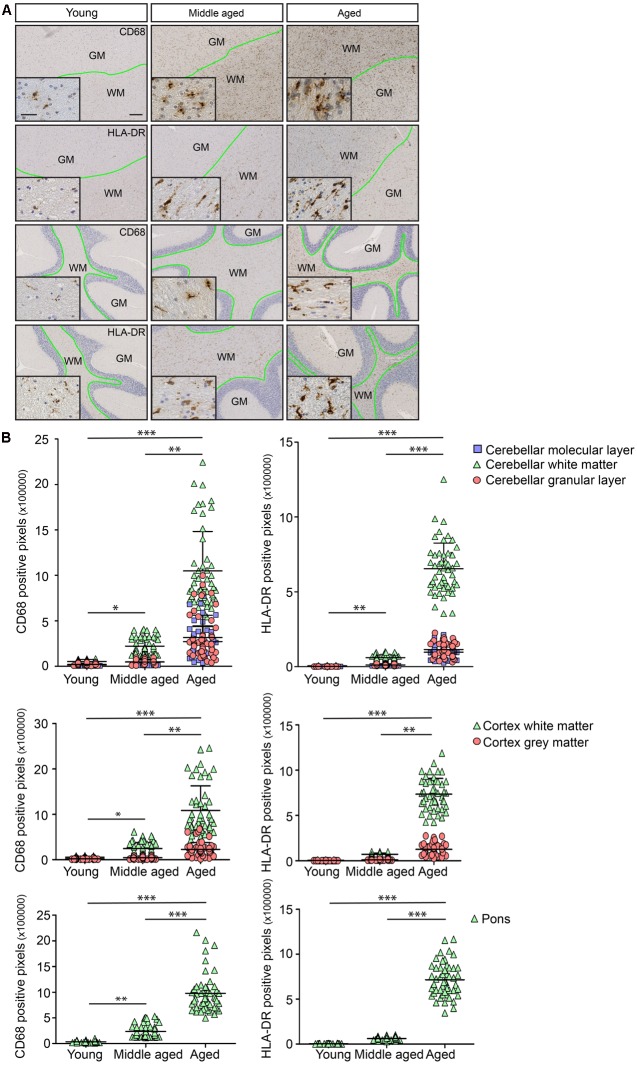
Age-associated immunoreactivity increases in CD68 and HLA-DR. **(A)** Immunostaining for CD68 and HLA-DR in young, middle aged, and aged human brain. The regions are cortex and cerebellum. The boundary between white and gray matter regions is drawn in green. **(B)** Quantification of immunostaining for CD68 and HLA-DR shows clear differences in white matter compared to corresponding gray matter within the same brain region. Changes in white matter start in middle aged samples (Young, *n* = 3; Middle aged, *n* = 6; Aged, *n* = 6). Scale bar: **(A)** = 200 μm, insert = 25 μm. Mann–Whitney’s test ^∗^*P* < 0.05, ^∗∗^*P* < 0.005, ^∗∗∗^*P* < 0.0005. Error bars indicate standard deviation (SD).

In order to investigate whether the observed differences in white- and gray matter in terms of morphology and gene expression in mice also occurred in humans, post-mortem human brain tissues of young (27–33 years), middle-aged (51–56 years), and aged (70–87 years) people were analyzed. The selected individuals were devoid of neurological conditions or inflammatory diseases around the period of death. Immunostaining for IBA1, CD45, HLA-DR, and CD68 was performed. Microglia stained with IBA1 and CD45 were found in white and gray matter (data not shown). CD68, a protein associated with lysosomes, was selectively detected in microglia in white matter regions including corpus callosum, cerebellar white matter, and cortical white matter (Supplementary Figures 2C,G,I compared to B,H; low magnification overview in supplementary Figure 2A). HLA-DR, a molecule involved in antigen presentation, was restricted to white matter tracts within the same brain regions (Supplementary Figures 2F,J,L compared to E,K; Low magnification overview in Supplementary Figure 2D). The expression levels of CD68 and HLA-DR increased with age in cortical and cerebellar white matter (**Figure 4A**). Quantification of immunostaining showed an increase with age and regional microglia activation (**Figure 4B**).

**FIGURE 5:**
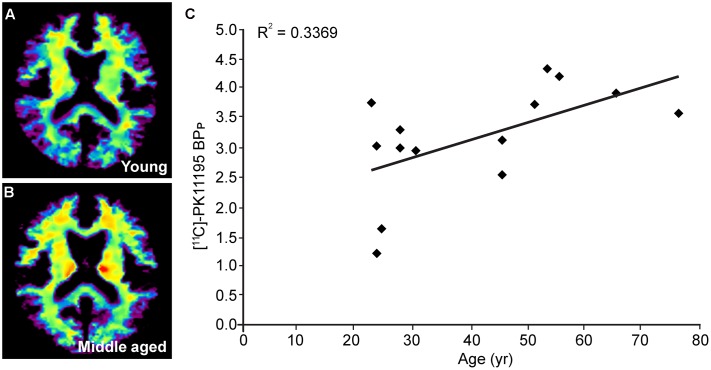
PET studies using [^11^C]-PK11195 to localize age-associated microglial activation in white matter. Axial image of the human brain showing white matter [^11^C]-PK11195 average uptake in **(A)** young and **(B)** middle aged healthy volunteers (n = 7); **(C)** Positive correlation between [^11^C]-PK11195 binding potential (BP_P_) in the total corpus callosum and age of subjects.

To further investigate if the regional differences in microglia activation can be observed non-invasively we quantified the extent of radioactive ligand [^11^C]-PK11195 binding using PET imaging. It must be noted, however, that [^11^C]-PK11195 is also known to bind reactive astrocytes and endothelium (Turkheimer et al., 2015). The PET images displayed an axial plane of the human brain, showing the binding of [^11^C]-PK11195 in the white matter of young and middle-aged healthy volunteers. The images (axial view) represent the average [^11^C]-PK11195 uptake in the white matter of seven young (**Figure 5A**) and seven middle-aged (**Figure 5B**) healthy volunteers 50–60 min post-injection. The axial images clearly showed average [^11^C]-PK11195 uptake increased in the white matter of the aged brain already during middle age. Positive correlation was observed in [^11^C]-PK11195 binding in the corpus callosum upon aging (**Figure 5C**). The binding of [^11^C]-PK11195, indicative of neuroinflammation, was significantly higher in the corpus callosum of middle-aged subjects than in young subjects (3.14 ± 0.51 vs. 2.31 ± 0.72; *p* < 0.05) (Details in **Table 6**).

**Table 6:** Binding potential of [^11^C]-PK11195 in different brain regions of young and mid-aged brains.

Brain region	Young average	Young SD	Middle-aged average	Middle-aged SD	*P*-value
Amygdala	1.42	0.35	1.79	0.37	0.090
Basal ganglia	1.26	0.21	1.65	0.73	0.238
Cerebellum	0.92	0.19	1.47	1.06	0.242
Cingulum	1.21	0.32	1.52	0.59	0.278
Frontal cortex	1.55	0.65	1.82	1.13	0.591
Hippocampus	1.14	0.29	1.47	0.39	0.094
Insula	1.04	0.13	1.19	0.29	0.228
Occipital cortex	1.68	0.81	1.36	0.28	0.381
Parietal cortex	1.72	0.88	1.28	0.22	0.262
Temporal cortex	1.05	0.28	1.21	0.21	0.279
Thalamus	1.04	0.27	1.16	0.42	0.566
Mesencephalon	1.51	0.51	2.05	1.23	0.307
Pons	1.31	0.37	1.89	0.59	0.051
Corpus callosum_total	2.69	0.92	3.64	0.63	0.045
Corpus callosum_trunk	2.31	0.72	3.14	0.51	0.038
Corpus callosum_splenium	3.52	1.16	4.34	1.23	0.223
Corpus callosum_genu	2.79	1.03	4.42	1.99	0.078
White matter	1.73	0.60	1.79	0.40	0.829

### Comparison of Microglia Activation upon Systemic Inflammation and LOAD

To evaluate the neuroinflammation status in aging and AD, we first investigated brain sections from people who died of acute inflammatory diseases, which were used as positive controls for the further study. In addition, we compared a young non-demented control group (Braak stage 0-I) and a group of LOAD tissue samples (Braak stage V-VI). For each group, we compared the expression of IBA1, HLA-DR, and CD68 between the patients who died of acute inflammatory disease and those who died without systemic inflammation (Supplementary Figure 3A). In the non-demented group, white-matter microglia of samples with acute infection showed deramified morphology (Supplementary Figure 3A) and expressed significantly higher levels of IBA1 and CD68. However, the expression of HLA-DR did not change significantly in the white matter between acute-inflammation- and non-inflammation groups (Supplementary Figure 3B). In the LOAD group, the expression of IBA1, HLA-DR, and CD68 was not significantly different between inflammation- and non-inflammation groups (Supplementary Figure 3B). In conclusion, these data indicate that acute inflammation in addition to neurodegeneration did not further alter microglial morphology.

### Increased Microglial Immunoreactivity in the White Matter of Early-Onset AD Brains

**FIGURE 6:**
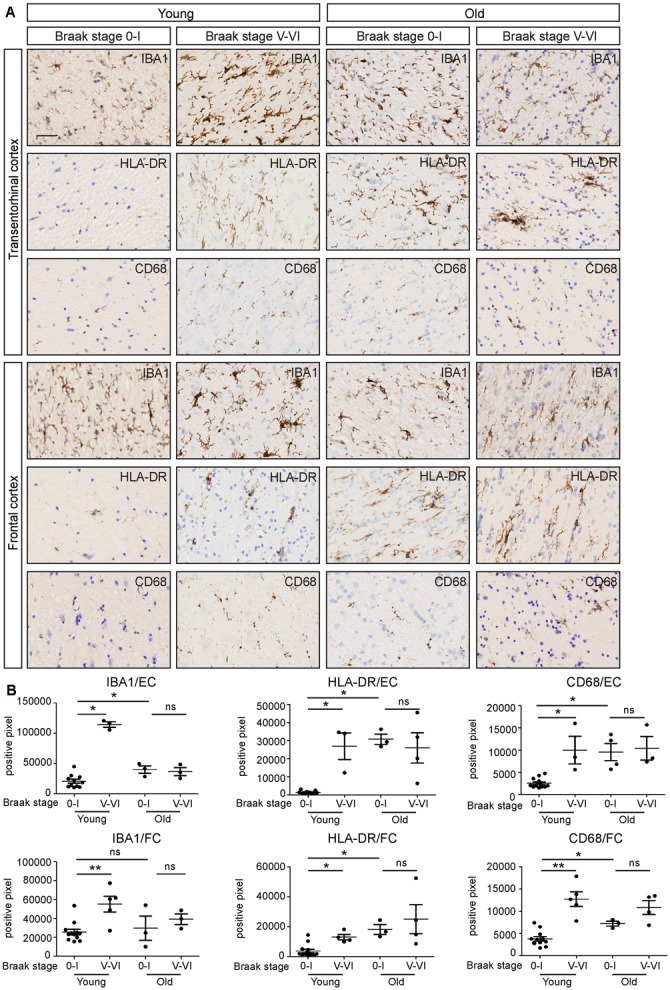
White matter immunoreactivity in IBA1, HLA-DR and CD68 increases in early-onset AD, but not in late-onset AD. **(A)** Transentorhinal cortex (EC) and frontal cortex (FC) sections of early-onset AD (Braak stage V-VI, *n* = 5), late-onset AD (Braak stage V-VI, *n* = 4) and their age-matched controls (Braak stage 0-I; Young controls *n* = 13; Old controls *n* = 3) are immunostained for IBA1, HLA-DR, and CD68. **(B)** Quantification of positive pixels of IBA1, HLA-DR, and CD68 staining shows that the expression of IBA1, HLA-DR, and CD68 increases in early-onset AD, compared to age-matched controls. There is no significant differences of IBA1, HLA-DR, or CD68 expression between the late-onset AD and age-matched controls. Scale bar: **(A)** = 40 μm. Mann–Whitney test, ^∗^*P* < 0.05, ^∗∗^*P* < 0.01, Error bars indicate standard error.

To investigate the effect of AD pathology on microglial immunoreactivity in white matter in relation to age, we compared the immunostaining between EOAD, LOAD, and age-matched controls (**Figure 6A**). Patients with inflammatory diseases prior to death were excluded. We investigated microglial activity in the white matter of FC and entorhinal cortex. Interestingly, white matter microglia in both regions of EOAD tissues showed significantly increased expression of IBA1, HLA-DR, and CD68, compared to age-matched controls (**Figure 6B**). To check the effect of aging on microglia activity, we studied the white matter of old controls, which showed upregulated expression of IBA1, HLA-DR, and CD68, compared to young controls (**Figure 6B**). To investigate the combined effect of aging and AD pathology on white matter pathology, we further compared the expression of IBA1, HLA-DR, and CD68 between LOAD and old controls. Interestingly, there was no significant difference between these two groups (**Figure 6B**). In summary, aging and AD-related pathology may increase microglia activity separately, but the combined effect is not additive. Aging- and AD-related microglial activation are morphologically similar.

## Discussion

In this study, we investigated age- and AD-related neuroinflammation in white matter tissue of both mice and humans. Upon aging, microglia activation (an indicator of neuroinflammation) was increased specifically in white matter regions in both mice and humans. The presence of activated microglia during aging in humans was also examined using PET scanning. The findings reveal that white matter is strongly affected both in aging and AD pathology. The regional phenotypic change of microglia in white matter may be of special relevance for the mechanism of brain aging. Whereas cognitive dysfunction during aging has been proposed to be due to neuronal loss, modern stereotactic approaches have shown limited neuronal death in the aged brain (Burke and Barnes, 2006; Rock and Kono, 2008; Bishop et al., 2010). The white matter tracts in the brain serve for functional connectivity and speed of processing. Compromised white matter integrity during aging can, therefore, affect cognition markedly (de Lange et al., 2016). Microglia as sentinels of the brain show early alterations in white matter, and therefore draw attention to changes in the white matter of the aging brain. In AD patients, the upregulation of proinflammatory cytokines, chemokines, and other immune mediators has been associated with neuropathology (Griffin et al., 1989; Xia et al., 1998; Ojala et al., 2009). Interestingly, the increased expression of immune factors may precede AD-like pathology (Krstic et al., 2012). Our research, for the first time, shows increased microglia-induced neuroinflammation in the white matter of EOAD, compared to age-matched controls. Interestingly, the microglia-induced neuroinflammation did not increase in the white matter of LOAD compared to age-matched controls.

We performed mRNA expression analysis of microglia isolated from young and aged mouse brains. As expected for aging brain tissue (Luo et al., 2010), the expression of pro-inflammatory genes involved in antigen presentation, interferon-, cytokine-, and chemokine signaling, and phagocytosis were enhanced in aged microglia. This gene expression pattern suggests that, during aging, the brain changes from a state of homeostatic equilibrium to a proinflammatory state. However, the activation of microglia is a graded process depending on the nature and extent of injury. The aging-related phenotype of microglia correlates with enhanced sensitivity for proinflammatory stimuli referred to as “immune primed” (Perry et al., 2007). Our data shows that the upregulation of pro-inflammatory transcripts is present at the RNA level, but not at the protein level. This suggests that, while microglia are in a “prepared” state at the RNA level, active translation or functional consequence is not initiated in the absence of an additional stimulus.

Several phagocytic receptors, including CD36, CD16/32, Axl, Dectin 1, and Trem2 are upregulated in white matter microglia. Scavenger receptor CD36 is involved in inflammatory responses to many ligands [e.g., lipids, amyloid β, and bacteria (Silverstein et al., 2010)]. Fc gamma receptors (i.e., CD16 and CD32) show multiple functions in a number of cellular responses including the release of inflammatory mediators, cytotoxic triggering, cell activation, and the phagocytosis of antibody-coated particles (Nagarajan et al., 1995). Axl is an inflammatory response receptor involved in the clearance of apoptotic cells, and its expression is induced by proinflammatory stimuli (Zagorska et al., 2014). Dectin 1 is a pattern-recognition receptor which can detect β-glucans in fungal cell walls (Goodridge et al., 2011). Trem2-mediated signaling has been shown to be responsible for regulating phagocytosis and lipid catabolism *in vivo* (Poliani et al., 2015). A rare R47H mutation of TREM2 correlates with a substantial increase in the risk of developing Alzheimer’s disease (AD) possibly due to impaired detection of damage-associated lipid patterns associated with neurodegeneration by microglia (wang et al., 2015). In our study, acute isolation of microglia from young and aged mouse brains shows that aged microglia phagocytose at a faster rate than young microglia, although the maximal amount of particles that can be phagocytosed by young and aged microglia is similar. Interestingly, other studies have convincingly shown that in aged microglia the phagocytosis of amyloid-β is impaired (Floden and Combs, 2011; Njie et al., 2012). Studies which investigated phagocytosis *in vivo* have shown exciting new functions for microglia, and this approach might reveal interesting insights for aging microglia as well (Sierra et al., 2013).

In line with previous findings, we observed that in human white matter, the activation of microglia was already detected at middle age (Salat et al., 2005; Brickman et al., 2006). A large gene expression study in the aging human brain also showed upregulation of neuroinflammation-associated genes at middle age (Lu et al., 2004). Interestingly, cognitive decline in humans has been reported to begin as early as at the age of 45 years (Singh-Manoux et al., 2012). PET imaging using the radioactive ligand [^11^C]-PK11195 for translocator protein (TSPO) located on the outer mitochondrial membrane of glial cells (Casellas et al., 2002), has been used to study neuroinflammation in a variety of conditions (Stephenson et al., 1995; Vowinckel et al., 1997; Venneti et al., 2009). In the current study, we investigated young and middle-aged subjects, but a systematic investigation in a large extended cohort of aged human subjects above 75 years old is required to fully assess whether we can effectively use this method especially in conjunction with approaches such as diffusion tensor imaging of white matter integrity to improve our diagnostic potential for neurodegenerative conditions. Currently, a proper correlation between the cognitive performance, and the BP_P_ in the white matter of middle-aged individuals is still lacking. A longitudinal study with repeated measurements of cognitive performance and BP_P_ of [^11^C]-PK11195 in different regions of the middle-aged control brain would be of interest in this matter.

Our study shows increased microglia-induced neuroinflammation in the white matter of EOAD, compared to young controls. Previous imaging studies showed white matter atrophy and degeneration in EOAD patients in the genu, body, and the splenium of corpus callosum, fornix, and main anterior-posterior pathways (Migliaccio et al., 2012; Caso et al., 2015), which is consistent with the white matter pathology observed in our study using immunohistochemistry. Interestingly, in a study using diffusion-tensor MR imaging subtle changes along the white matter tracts in AD patients were detected (Caso et al., 2015). This would mean that white matter abnormalities could be an early marker that possibly precedes gray matter atrophy, particularly in EOAD (Caso et al., 2015), as well as in healthy aging. Previous studies in mild cognitive impairment patients clearly indicated that white matter changes could be detected even before the development of cortical atrophy (Agosta et al., 2011; Maier-Hein et al., 2015). Detection of neuroinflammation at an early stage might serve as a good predictor for the onset of dementia and neurodegeneration in EOAD. Clearly, our present study using immunohistochemistry to investigate the post-mortem samples has limitations to answer whether the occurrence of neuroinflammation in the white matter is indeed the initiator of the cognitive decline in EOAD. To address this question in future, it will be necessary to conduct a long-term study (including neuroimaging and cognitive studies) on the carriers of EOAD mutations at the preclinical stage.

In contrast to EOAD, the expression of IBA1, HLA-DR, or CD68 in the white matter of LOAD brain is not significantly higher than that in age-matched controls. The different underlying mechanisms of EOAD and LOAD may explain the difference in the contribution of the neuroimmune system to AD pathology. Early-onset familial AD patients carry mutations in three genes, which encode amyloid precursor protein (APP), presenilin-1 (PS1), and PS2. These mutations can change the APP metabolism profoundly, and lead to overexpression and aggregation of Aβ. However, in LOAD, recent genome-wide association studies have found risk genes (e.g., *TREM2*, *CD33*) relating to microglia phagocytosis in the innate immune system. The mutations of *TREM2* and *CD33* will lead to reduced Aβ clearance and an increased inflammatory response (Griciuc et al., 2013; Hickman and El Khoury, 2014). The chronic inflammatory response may cause the dysregulation of clearing misfolded neuronal proteins which accumulate during aging (Krstic and Knuesel, 2013). The relationship between white matter changes and cognitive functions in LOAD is a matter of debate, and neuroimaging studies have shown conflicting results. A few studies have reported a significant correlation (Diaz et al., 1991; Stout et al., 1996), whereas others have failed to find any relationship in AD (Leys et al., 1990; Brilliant et al., 1995; Hirono et al., 2000). In our study, the non-significant change in the white matter of LOAD and aging brains supports the latter notion that cognitive decline in LOAD is not associated with white matter change.

Until now, the systematic evaluation of white matter neuroinflammation in relation to aging and neurodegeneration has received much less attention than gray matter neuroinflammation. Postmortem AD brain samples analysis, together with the information of premortem cognitive status and the presence of neuritic plaques, has shown that microglia activation is more prominent in white matter than in gray matter at early clinical dementia stages (i.e., CDR score: 0, non-demented; 0.5, questionable dementia) (Xiang et al., 2006). With progressive increase in plaque pathology and the worsening of clinical dementia symptoms, microglia activation becomes comparable in both white- and gray matter (Xiang et al., 2006). Here, we provide evidence of increased microglia-induced neuroinflammation in the white matter of aging and AD brains. With the fast development of neuroimaging techniques and novel imaging-based, CSF-based biomarkers (Humpel, 2011; Varrone and Nordberg, 2015; Zhang, 2015), the detection of microglia-induced neuroinflammation in the white matter may identify reliable markers to monitor disease onset, progression, and therapeutic efficacy.

## Author Contributions

DR designed and conducted animal experiments. DR and ZY designed and conducted experiments on human samples. DR and ZY analyzed the data. ZY finalized the figures. MB and IM-O provided the technique support for immunohistochemistry. JD conducted the PET experiment. IH helped to conduct the statistical analysis. MO conducted the phagocytosis assay. DVD and PDD provided EOAD and LOAD human samples. WdD and SA provided human samples and helped to supervise the experiments using human samples. BE helped to design the experiments. ZY and DR wrote the manuscript. EB conceived, supervised, and provided funding for the study.

## Conflict of Interest Statement

The authors declare that the research was conducted in the absence of any commercial or financial relationships that could be construed as a potential conflict of interest.

## Abbreviations

AD: Alzheimer’s disease
BP_P_: binding potential
CDR: clinical dementia rating
CNS: central nervous system
CRP: C-reactive protein
EOAD: early-onset Alzheimer’s disease
FACS: fluorescence-activated cell sorting
FDR: false discovery rate
GO: gene ontology
LOAD: late-onset Alzheimer’s disease
NFT: neurofibrillary tangles
PET: positron emission tomography
PFA: paraformaldehyde
ROIs: regions of interest
SPM: Statistical Parametric Mapping.
