# Supplementary material for: Increased White Matter Inflammation in Aging- and Alzheimer’s Disease Brain
Source: Front Mol Neurosci. 2017 Jun 30;10:206. doi: 10.3389/fnmol.2017.00206 (PMC5492660; doi:10.3389/fnmol.2017.00206)
Supplement: Supplementary file 1 [file Data_Sheet_1.PDF]

Supplementary materials for

Increased white matter inflammation in aging- and Alzheimer's disease brain

Divya Raj*, Zhuoran Yin*, Marjolein Breur, Janine Doorduin, Inge R. Holtman, Marta Olah, Ietje J. Mantingh-Otter, Debby Van Dam, Peter P. De Deyn, Wilfred den Dunnen, Bart J. L. Eggen, Sandra Amor, Hendrikus W.G.M. Boddeke

* These authors contributed equally to this work.

Supplementary figures

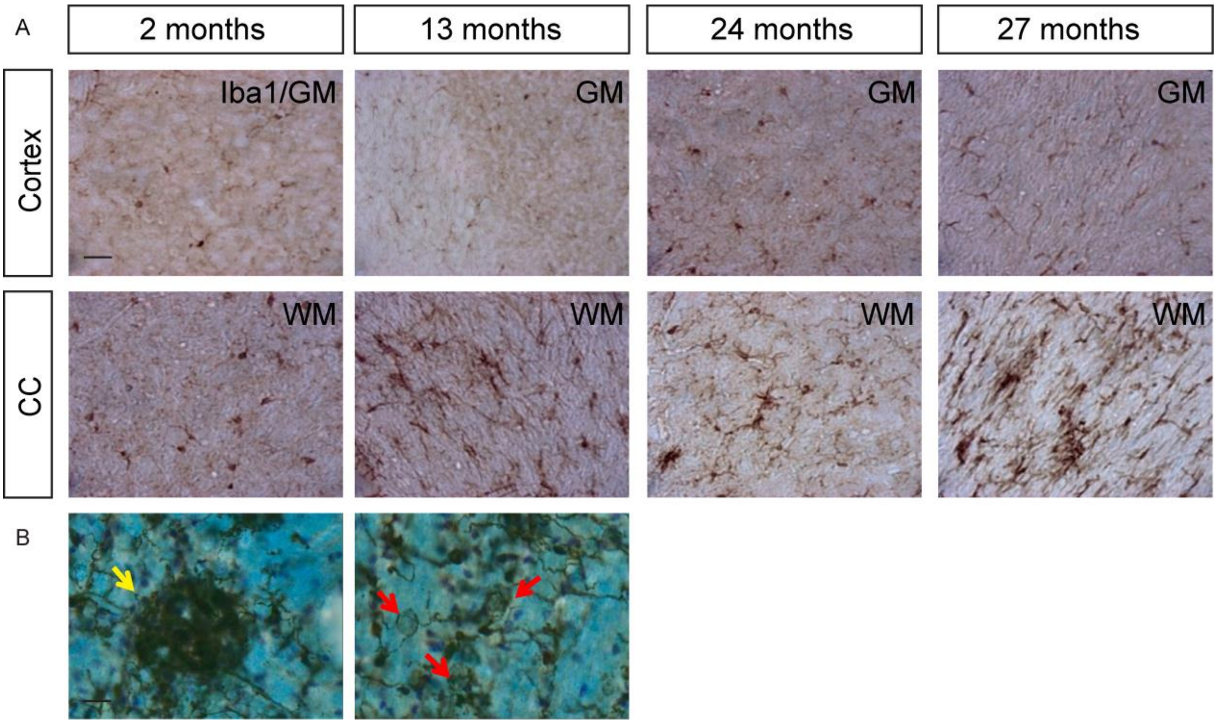

Supplementary Figure 1 Increased expression of Iba1 in the white matter during aging. **(A)** Immunostaining of Iba1 showed changes in microglia morphology with increasing age (2 months; 13 months; 24 months; 27 months) in gray matter (GM) of cerebral cortex and white matter (WM) of corpus callosum (CC) (n = 3). White matter microglia showed progressive changes from age 13 months, while gray matter microglia begin to change at age 24 months; **(B)** Iba1 staining showed clusters of microglia in the aged brain, while such clustering is absent in young brain. Spheroids frequent in processes of aged microglia in the white matter visualized with Iba1 staining; Scale bar: **A** = 100 µm, **B** = 50 µm.

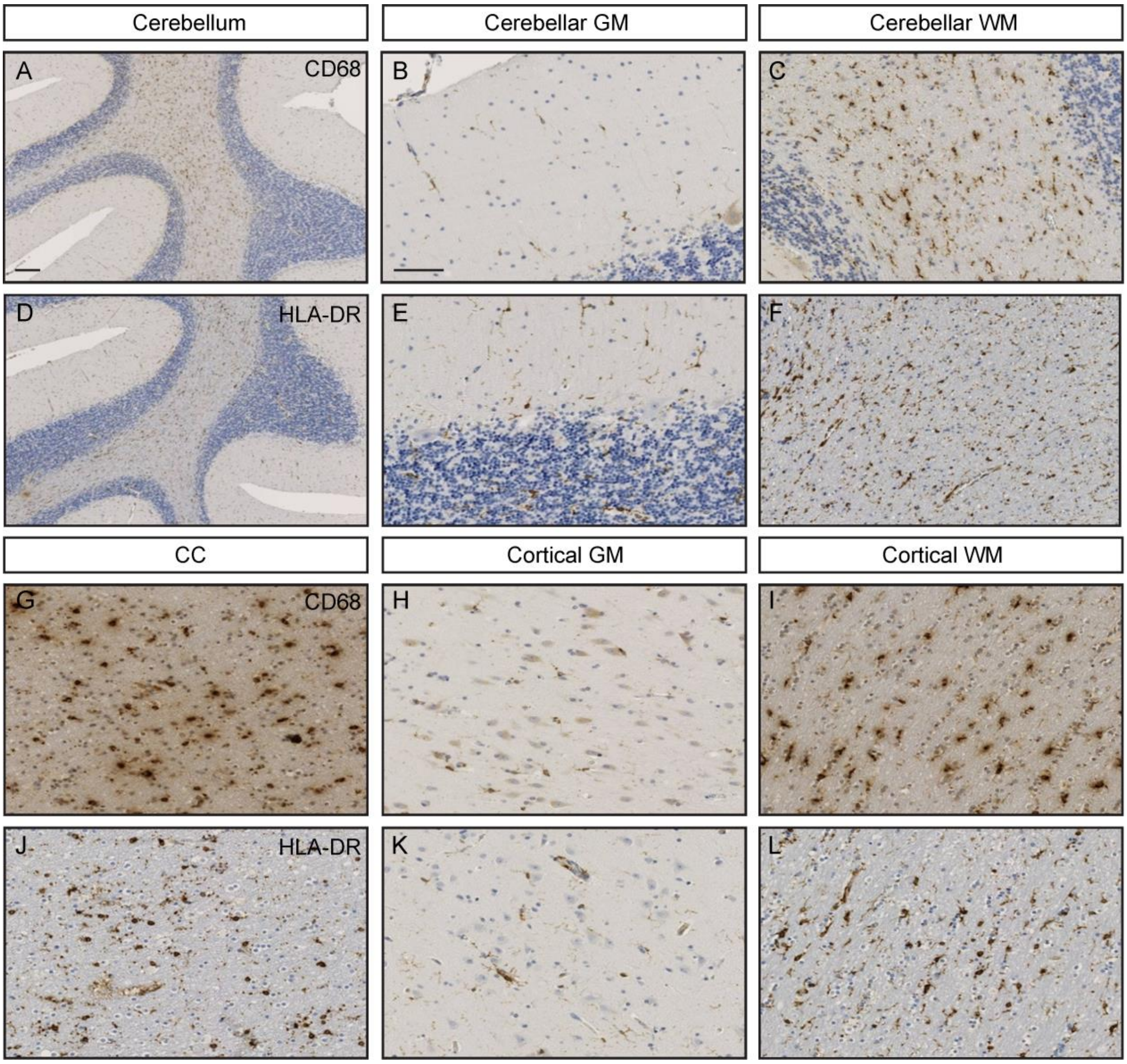

Supplementary Figure 2 Immunohistochemical staining with CD68 and HLA-DR in the aged brain. Representative immunohistochemical staining for CD68 (**A**-**F**) and HLA–DR (**G**-**L**) in the cortex, cerebellum, and corpus callosum (CC) of aged brain. Increased staining for CD68 in white matter compared to gray matter (**c** compared to **B**; **G**, **I** compared to **H**). Increased staining for HLA-DR in white matter compared to gray matter (**F** compared to **E**; **J**, **L** compared to **K**). Scale bars: **A**, **G** = 200 μm; **B**-**F**, **H**-**L** = 100 μm.

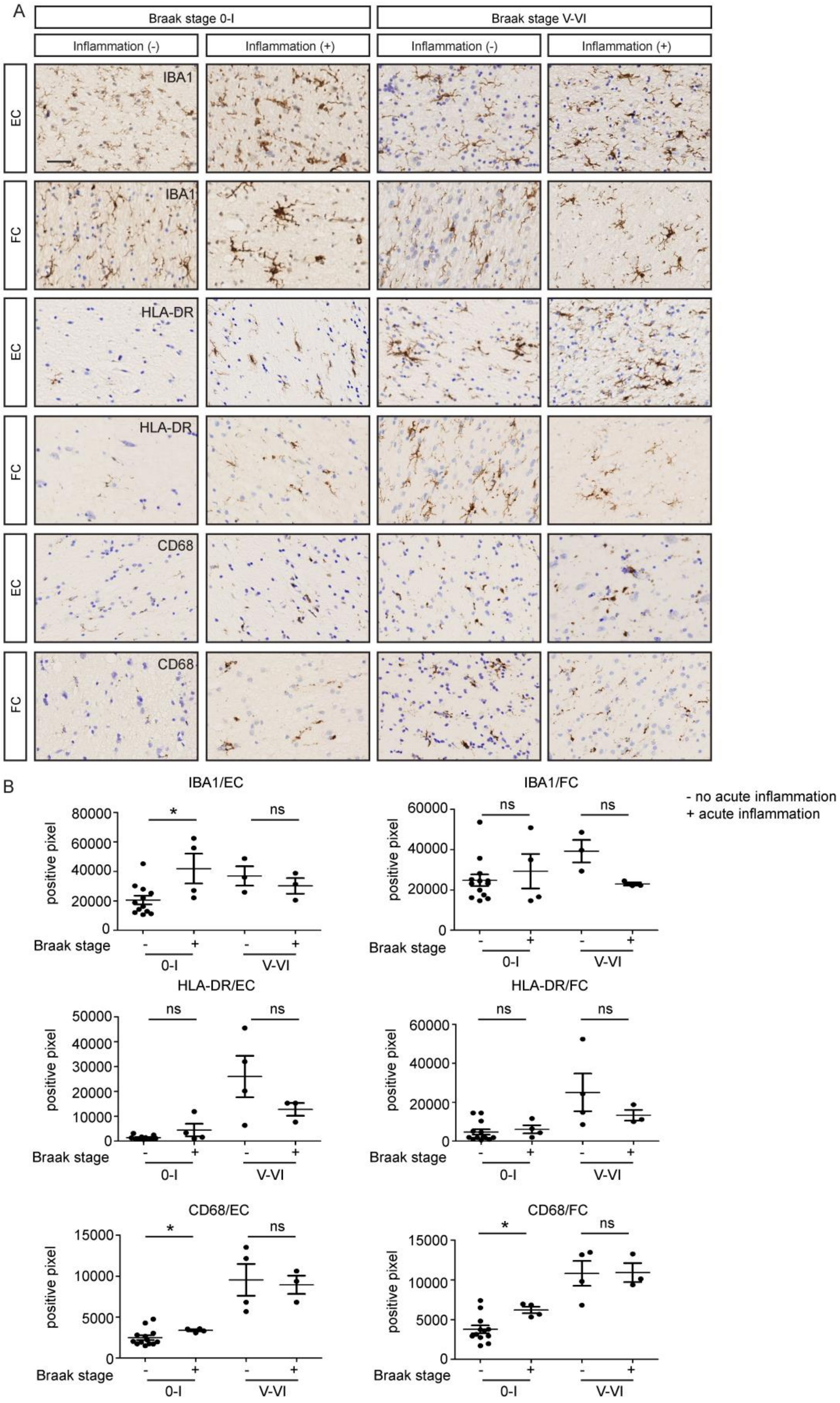

Supplementary Figure 3 Systemic inflammation increases the expression of IBA1 and CD68 in the white matter of non-demented brain. In order to detect neuroinflammation, brain

sections of patients who died of acute inflammatory diseases were applied as positive controls. **(A)** Transentorhinal cortex (EC) sections and frontal cortex (FC) of non-demented subjects (Braak stage 0-I) and late-onset AD patients with/without acute systemic inflammation were immunostained for IBA1, HLA-DR, and CD68. **(B)** The positive pixels of IBA1, HLA-DR, and CD68 staining in the white matter were compared between subjects with systemic inflammation and those without systemic inflammation. In non-demented subjects (Braak stage 0-I), IBA1 and CD68 increased in patients with systemic inflammation. The difference in HLA-DR expression between subjects with and without systemic inflammation did not reach significance. In the late-onset AD group, no significant differences of IBA1, HLA-DR or CD68 expression between subjects with and without systemic inflammation were noted (Nonparametric test: Mann-Whitney test, *$p < 0.05$, mean ± s.e.m). Scale bar: **A** = 40 μm.
